# 肺部多发磨玻璃影的外科治疗

**DOI:** 10.3779/j.issn.1009-3419.2016.06.11

**Published:** 2016-06-20

**Authors:** 群 王, 伟 蒋, 俊杰 奚

**Affiliations:** 200032 上海，复旦大学附属中山医院胸外科 Department of Thoracic Surgery, Zhongshan Hodpital, Fudan University, Shanghai 200032, China

**Keywords:** 肺肿瘤, 肺部磨玻璃影, 手术, Lung neoplasms, Pulmonary ground glass opacity, Surgery

## Abstract

肺部磨玻璃影（ground glass opacity, GGO）的发病率近年来日益增高，很多患者发现有多发GGO，但多发GGO的诊疗还存在争议。肺部GGO是一种影像学表现，包含了多种病理类型，其中有一部分GGO是早期肺癌。GGO是一种惰性结节，只有少数GGO会发生变化，且随访不会影响外科治疗的效果。多发GGO的手术时机主要由主病灶决定，主病灶中实性成分大于5 mm时建议外科干预。手术方式可以选择肺叶切除或亚肺叶切除，除主病灶以外，其他GGO不必全部切除。具有高危因素的多发GGO需要纵隔淋巴结清扫或采样。

## Abstract (en)

The incidence of pulmonary ground glass opacity (GGO) has been increasing in recent years, with a great number of patients having multiple GGOs. Unfortunately, the management of multiple GGOs is still controversial. Pulmonary GGO is a radiological term, consisting of different pathological types. Some of the GGOs are early-staged lung cancer. GGO is an indolent nodule, only a small proportion of GGOs change during observation, which does not influence the efficacy of surgery.. The timing of surgery for multiple GGOs mainly depends on the predominant nodule and surgery is recommended if the solid component of the predominant nodule > 5 mm. Either lobectomy or sub-lobectomy is feasible. GGOs other than the predominant nodule can be left unresected. Multiple GGOs with high risk factors need mediastinal lymph node dissection or sampling.

肺部磨玻璃影（ground glass opacity, GGO）是指肺内局灶性结节样密度增高影，但其密度又不足以掩盖在其中走行的支气管血管束^[1, 2]^。根据其内是否含实性成分，分为纯磨玻璃影（纯GGO）和部分实性磨玻璃影（部分实性GGO）。肺内磨玻璃影数量≥2个时称为多发磨玻璃影。随着影像学技术的进步和人们健康意识的提高，GGO的发病率日益增高，国内外学者对于肺部GGO，尤其是多发性GGO的诊治还存在争议。本文就肺部多发GGO的诊疗现况进行综述。

## 1 肺部GGO的生物学特性

肺部GGO是影像学术语，包含了恶性肿瘤、良性肿瘤、炎症、肺间质性疾病、肺内淋巴结等病理类型。许多研究结果表明GGO的影像学表现和病理关系密切^[3-5]^。纯GGO是GGO形成的初期，而部分实性GGO则是GGO的后期形态。2011年国际肺癌研究协会（The International Association for the Study of Lung Cancer, IASLC）、美国胸科学会（American Thoracic Society, ATS）、欧洲呼吸学会（European Respiratory Society, ERS）联合公布了新的肺腺癌的国际多学科分类标准^[6]^，其主要包括：不典型腺瘤样增生（atypical adenomatous hyperplasia, AAH）、原位腺癌（adenocarcinoma *in situ*, AIS）、微浸润腺癌（minimally invasiveadenocarcinoma, MIA）、浸润性腺癌及浸润性粘液腺癌。这一新的分类对基于计算机断层扫描（computed tomography, CT）表现的治疗指南的形成具有直接指导意义。纯GGO病理多为AAH、AIS和MIA，而部分实性GGO病理则以MIA和浸润性腺癌居多。有研究^[7]^显示长期存在的小的纯GGO，尤其是直径 < 5 mm的纯GGO，常被病理证实为AAH。而AIS和MIA这两类患者若接受根治性手术，其5年无病生存率可达到100%或接近100%。GGO中实性成分含量越多，则预后相对越差^[8, 9]^。

筛查中发现的一部分GGO会在随访过程中消失，而持续存在的GGO中绝大多数也变化缓慢。韩国于1997年至2006年采用低剂量CT（low dose CT, LDCT）在正常人群中筛查了19, 919例，其中被发现且持续存在2年以上的纯GGO共122个。研究中位随访时间为59个月，90.2%的GGO没有变化或缩小，12例增大的GGO中位体积倍增时间为769 d。11例接受了手术切除，病理为2例AIS、6例MIA和3例浸润性腺癌^[10]^。

日本学者进一步将部分实性GGO细分成两类进行了研究。一项日本国立癌中心牵头的前瞻性临床试验将结节最大径≤3 cm，实性成分≤5 mm的GGO纳入研究^[11]^，并根据其在CT肺窗和纵隔窗的表现分为三种类型：①纯GGO，在肺窗及纵隔窗均未见实性成分；②异质性GGO，仅在肺窗可见实性成分；③部分实性GGO，在肺窗和纵隔窗均见实性成分。其最终共纳入1, 046例纯GGO，81例异质性GGO，102例部分实性GGO。经过平均（4.3±2.5）年的随访，1, 046例纯GGO中13例（1.2%）发展为异质性GGO，56例（5.4%）发展为部分实性GGO，发生改变的时间平均为发现后的（3.8±2.0）年。81例异质性GGO中16例（19.8%）发展为部分实性GGO，平均需要（2.1±2.3）年。35例纯GGO接受了手术切除，病理为5例AAH、21例AIS和9例MIA。7例异质性GGO接受了手术切除，病理为2例AIS和5例MIA。部分实性GGO中49例接受手术切除，病例为1例AAH、10例AIS、26例MIA和12例浸润性腺癌。这项研究表明GGO的发展过程依次是纯GGO、肺窗可见实体成分、肺窗及纵隔窗均可见实体成分，并且这一发展的过程极其缓慢。

## 2 多发性GGO进一步处理的时机

鉴于GGO是一种惰性的结节，目前的临床指南不建议对多发性GGO采取过于积极的治疗方案。Fleischner学会^[12]^2013年发表的肺非实性结节处理指南中，对于多发性GGO的随访治疗提出了建议：如果多发GGO均为纯GGO且没有突出病灶，则继续随访；如果多发GGO中有突出的实性结节，则在首次检查后3个月进行CT随访证实病灶存在，如果病变持续存在，推荐活检或外科治疗，尤其是对内部实性成分直径 > 5 mm的病灶。而2016年的NCCN肺癌筛查指南中，对于多发性GGO也有类似的随访流程建议。Fleischner学会^[12]^认为突出病灶包括：部分实性特别是那些实性成分 > 5 mm的GGO； > 10 mm的纯GGO；具有毛刺轮廓、空泡征或网格征的不典型的部分实性结节；纯GGO或内部实性成分 < 5 mm的部分实性结节，若随访过程中出现病灶大小或密度的变化均要高度怀疑为恶性。

多发GGO主病灶的最大径和实性成分最大径/结节最大径比值（C/T值）都是医师判断结节良恶性和手术时机的参考依据。Kim等^[13]^回顾了40例手术切除的纯GGO，发现 < 5 mm的纯GGO均为良性结节，而5 mm-10 mm的纯GGO中，仅10.5%是肺恶性肿瘤。作受试者工作特征曲线（receiver operating characteristic curve，ROC曲线）进行分析后发现8 mm可以作为纯GGO是否继续随访的临界值。日本的前瞻性多中心临床试验JCOG0201^[14]^共纳入了545例≤3 cm的外周型GGO，分析发现最大径 < 2 cm且C/T值< 25%的肿瘤均未侵犯淋巴管、血管或淋巴结，并将其命名为影像学范畴上的非侵袭性肿瘤。在随后的验证随访^[15]^中发现，非侵袭性肿瘤患者的5年生存率为97.1%。

## 3 正电子发射型计算机断层显像（positron emission computed tomography, PET）-CT对于多发GGO的诊断价值

PET-CT并非对于所有的肺部小结节都有诊断价值。小的纯GGO在PET上常常没有糖代谢增高，且很少发生淋巴结转移^[16-20]^。Kim等^[21]^对89例患者的134个GGO进行PET-CT检查，发现最大标准摄取值（maximum standard uptake value, SUVmax）与结节大小和C/T值正相关，79%的多发性GGO糖代谢没有增高，且淋巴结转移和远处转移的真阳性率为0，因此作者认为PET-CT对于GGO的术前诊断价值较低。据此，Fleischner学会认为对于纯GGO，PET-CT的诊断价值有限。而对于部分实性GGO，PET-CT有一定价值。Higashi等^[22]^发现在3 cm以下的肺腺癌中，CT上可见实性成分和PET糖代谢增高是不良预后因素。Pastorino等^[23]^也发现肺部结节PET-CT的糖代谢与患者预后密切相关，PET-CT能减少对于惰性肿瘤不必要的治疗。Fleischner学会^[12]^建议对于多发GGO，如果有突出病灶是8 mm-10 mm的部分实性GGO，则进行PET-CT进一步检查，有利于更准确地评估预后及优化术前分期。

## 4 多发GGO的外科切除

由于表现为GGO的肺癌是一种发展缓慢的肿瘤，一般多发GGO不被认为是肿瘤-淋巴结-转移（tumor-node-metastasis, TNM）分期中的T3或T4，而是被认为多个原发肿瘤。Gu等^[24]^回顾了39例接受手术的多发GGO，平均随访30.7个月后，患者的总生存率为100%。IASLC分期及预后委员会总结了相关文献，并对多发GGO的分期提出了建议^[25]^。该文建议对于多发GGO，T分期以最大的病灶作为标准，并在T分期后以括号表明肿瘤数量或仅标注（m）表示多发病灶。不管多发GGO发生在同一肺叶或是同侧不同肺叶或双侧肺，T分期都以此标注。

文献^[26-31]^报道中多发GGO患者接受手术切除后的预后均令人满意，即使是亚肺叶切除也不影响预后。Yao等^[27]^总结了2009年-2014年29例同期双侧肺结节切除术，其中切除了26个纯GGO和20个部分实性GGO。其中1例行双侧肺叶切除，16例行肺叶+亚肺叶切除，12例行双侧肺叶切除，术后均无重大并发症，经过10个月-51个月的随访，未发现死亡或复发。

虽然发生多发GGO的病因现在仍不明确，但多发GGO的患者可能较其他人更容易产生新的GGO。Mun等^[28]^回顾了2000年-2006年27例手术切除的表现为GGO的细支气管肺泡癌，共91个GGO。经过平均46个月的随访，所有患者均存活，但7例出现了新发病灶，并认为新发病灶是治疗多发GGO中的问题。

对于多发GGO，外科医师还面临一个问题：是否应当将所有的GGO都同期或分期切除。Shimada等^[29]^回顾了67例至少有1个GGO的多发结节，结果发现主病灶C/T值≤0.5的患者5年生存率为95.8%，而另一组患者5年生存率为68%，多因素分析提示主病灶大和C/T值> 0.5都是不良的预后因素。更重要的发现是，当切除主病灶后，无论剩余的GGO病灶继续生长，还是出现新的GGO病灶，或剩余的GGO病灶未予处理，都不会影响患者的总生存率。Kim等^[32]^回顾了73例手术切除的细支气管肺泡癌，其中23例为多发纯GGO。多发纯GGO中有18例未将GGO全部切除，经过平均40.3个月的随访，15例剩余的GGO没有变化，3例剩余的GGO消失，所以认为没有必要切除所有的GGO。

## 5 多发性GGO的淋巴结清扫

多发性GGO手术切除后是否需要纵隔淋巴结清扫/采样也是胸外科医生关注的问题之一。Shimada等^[29]^对于67例多发GGO的外科治疗中，59例接受了纵隔淋巴结切除，包括10例系统性淋巴结清扫、33例选择性淋巴结清扫和16例淋巴结采样，对于C/T值≤0.5的病例，随访后均无复发转移。Yao等^[27]^治疗的29例双侧肺结节中，对其中的18例采取了系统性淋巴结清扫。

多发性GGO主病灶的C/T值被认为与淋巴结转移密切相关，能帮助医师选择是否进行淋巴结切除及切除的方式。Haruki等^[33]^回顾了876例临床Ⅰ期肺癌，患者均接受了系统性淋巴结清扫，分析发现133例C/T值< 0.5的肿瘤没有肺门或纵隔淋巴结转移，而C/T值≥0.5的肿瘤中有12%发生了淋巴结转移。Ye等^[34]^回顾了651例Ⅰa期肺腺癌，发现55例纯GGO中没有淋巴结转移，292例部分实性GGO中有6例发生了N1淋巴结转移，3例发生了N2淋巴结转移。另外还发现AAH、AIS、MIA、贴壁为主型腺癌和浸润性粘液腺癌没有淋巴结转移。Koike等^[35]^分析了894例Ⅰa期外周型肺癌，进行多因素分析后同样发现C/T值≥89%是淋巴结转移的预测因素。

## 6 展望

多发性GGO的切除适应症目前还没有统一的标准，随着个体化治疗和精准医疗的发展，多发性GGO的治疗也需要提高准确性和精确性，减少患者不必要的创伤。肺腺癌新分类的提出及其预后意义为淋巴结清扫提供了更多的参考依据，随着术中冰冻病理准确性的提高，术中对于淋巴结清扫的判断将更为准确。
